# Meta-Analysis of Oncological Outcome After Abdominoperineal Resection or Low Anterior Resection for Lower Rectal Cancer

**DOI:** 10.1007/s12253-014-9863-x

**Published:** 2014-11-28

**Authors:** Xiao-Tong Wang, De-Gang Li, Lei Li, Fan-Biao Kong, Li-Ming Pang, Wei Mai

**Affiliations:** 1 Departments of Gastrointestinal and Peripheral Vascular Surgery, People’s Hospital of Guangxi Zhuang Autonomous Region, Nanning, People’s Republic of China; 2 Departments of Surgery, The First Affiliated Hospital of Guangxi University of Chinese Medicine, Nanning, People’s Republic of China

**Keywords:** Low anterior resection, Abdominoperineal resection, Lower rectal cancer, Prognosis

## Abstract

In lower rectal cancer, postoperative outcome is still subject of controversy between the advocates of abdominoperineal resection (APR) and low anterior resection (LAR). Reports suggest that low anterior resection may be oncologically superior to abdominoperineal excision, although no good evidence exists to support this. Publications were identified which assessed the differences comparing 5-year survival, local recurrence, circumferential resection margin rate, complications and so on. A meta-analysis was performed to clarify the safety and feasibility of the two procedures with several types of outcome measures. A total of 13 studies met the inclusion criteria, and comprised 6,850 cases. Analysis of these data showed that LAR group was highly correlated with 5-year survival (pooled *OR* = 1.73, 95%CI: 1.30–2.29, *P* = 0.0002 random-effect). And local recurrence rate of APR group was significantly higher than that in LAR group (pooled *OR* = 0.63, 95%CI: 0.53–0.75, *P* < 0.00001 fixed-effect). Also, the circumferential resection margin (CRM) were high involved in APR group than in LAR group. (5 trials reported the data, pooled *OR* = 0.43, 95%CI: 0.36–0.52, *P* < 0.00001 fixed-effect). Besides, the incidents of overall complications of APR group was higher compared with LAR group (pooled *OR* = 0.52, 95%CI: 0.29–0.92, *P* = 0.03 random-effect). Patients treated by APR have a higher rate of CRM involvement, a higher local recurrence, and poorer prognosis than LAR. And there is evidence that in selected low rectal cancer patients, LAR can be used safely with a better oncological outcome than APR. due to the inherent limitations of the present study, for example, the trails available for this systematic review are limited and the finite retrospective data, future prospective randomized controlled trials will be useful to fully investigate these outcome measures and to confirm this conclusion.

## Background

Colorectal carcinoma is the fourth most common malignancy worldwide. The major sphincter-saving operation (SSO) low anterior resection (LAR) is more common than abdominoperineal resection (APR) in the treatment of rectal cancer. rectal cancer patients preferred LAR to APR as it avoided the requirement for a permanent stoma, despite the fact that LAR was associated with a risk of postoperative complications including fecal incontinence [1]. APR are therefore considered only where sphincter-preserving LAR are not feasible, or only when the tumor is fixed to the anal sphincter or is less than 1 cm above the anal sphincter [2]. Most studies have reported an APR to LAR ratio of 1:3 or 1:4 [3, 4], suggesting that LAR may be oncologically superior to APR [5]. A number of studies comparing the short-term or long-term outcomes, of LAR vs APR for lower rectal cancer have shown that LAR has become the preferred option in curative surgery [6–9]. Patients treated by APR have a higher rate of circumferential margin involvement, a higher local recurrence, and poorer prognosis than LAR [10–12]. However, most studies were too small to adequately evaluate the surgical outcomes. For those patients with lower rectal cancer eligible for surgical treatment, whether APR or LAR is the better choice remains controversial [13–15]. Therefore, this article has prompted the present comparison of the operative results and oncologic outcomes of those patients treated by LAR and APR. We performed a meta-analysis of all the studies directly comparing APR and LAR in the treatment of low rectal cancer. these results may help to determine that the selected surgical procedure, either LAR of APR, is performed safely.

## Materials and Methods

### Information Sources and Search

A systematic literature search was performed independently by two of the authors (FBK and XTW) using Medline, Embase, BioMed Central, CNKI (Chinese National Knowledge Infrastructure Database), Wangfang (Database of Chinese Ministry of Science & Technology), and CBM (China Biological Medicine Database) and performed on all studies for potentially relevant records comparing APR and LAR. The search was limited to humans. Animal trials were be excluded. No restriction was set for date of publication. FBK and XTW assessed titles or abstracts of all identified studies independently and exclude all the irrelevant ones. Full text articles of potentially relevant studies were obtained. These studies were assessed independently in an unblended standardized manner by FBK and XTW as to whether they met the inclusion criteria for this review.

Combinations of the following search terms were be used: “abdominoperineal resection”; “low anterior resection”; “colon” or “rectum”; “cancer”, “neoplasia” or “tumor”.

To minimize retrieval bias we performed a manual search method that utilize the Google Scholar database, Science Citation Index, Cochrane Library, and manually searched seven high-impact journals, chosen on the basis of the frequency of articles and on expert opinion. When further information was required, the corresponding authors of relevant papers were contacted by the authors (FBK and XTW). The reference lists of all potentially eligible studies will be reviewed. Researchers who may have carried out relevant studies will be contacted.

### Inclusion Criteria

The following criteria were fulfilled for the studies included in the meta-analysis: (1) trials had to be published as a full paper in English or Chinese language literature. (2) The studies compared the original outcomes of LAR and APR in the treatment of patients with low rectal cancer within 6 cm of the anal verge; (3) no preoperative radiotherapy, chemotherapy, and/or neoadjuvant chenoradiation administered to the patients; (4) the patients’ clinical and pathologic parameters (age, sex, tumor differentiation, TNM classification, margin status and so on) must be mentioned in article. (5) If more than one studies were reported by the same institute or author, only the most recent or the highest level of studies were included.

### Exclusion Criteria

The following studies were excluded: (1) The inclusion criteria were not met; (2) with operation contraindication; (3) The research samples were too small and the cases were less than 20 cases; (4) there was no initial data or cannot search original literatures; (5) the original studies only assessing outcome of either LAR or APR; (6) the patients accepted other treatment before or after surgery and these treatments could lead to distinct prognosis; (7) review articles, letters, comments, case reports.

### Data Extraction

Data extraction was performed independently by two of the authors (KFB and WXT). Disagreements were solved through discussion, if necessary, by involving an independent third author(LMP). The main extracted data included: (1) First author and the year of publication, (2) The number and characteristics of trial participants, (3) institution, study design, inclusion and exclusion criteria, matching criteria, sample size (cases and controls or cohort size). (4) The outcome of the trials including the 5-year survival and local recurrence plus overall complications. (5) We made an effort to contact all primary authors of studies by e-mail to standardize their data according to the meta-analysis definitions whenever possible, and in all cases of missing or incomplete data, the primary authors were contacted for original information, but none provided any additional information.

## Results

### Description of Eligible Studies

**Fig. 1:**
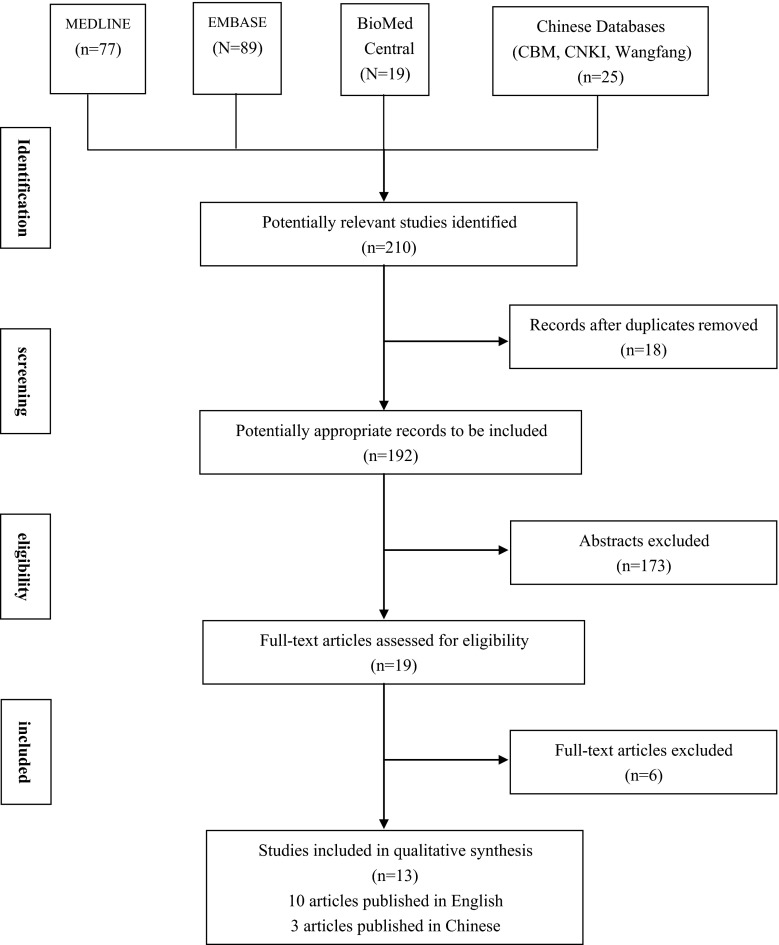
Flowchart of the search process

Using the search strategy described above and excluding duplicates, 192 publications were identified. One hundred and seventy three studies were excluded following title and abstract review. Five further studies were excluded after evaluation of the full-text in detail in full. This literature search identified 14 studies that compared the results of LAR and APR for rectal cancer. Of these, one study was excluded from the analysis because it did not provide adequate results for the outcomes of interest. This left 13 studies, which fulfilled the inclusion criteria and formed the basis of this review (Tables 1 and 2). On view of the data extraction, there was 100 % agreement among the three reviewers. These included a combined total of 6,850 subjects, of which 3,866 (56.44 %) underwent LAR and 2,984 (43.56 %) underwent APR as the primary operative intervention for rectal cancer. Two studies [6, 15] contained groups that were fully matched for age and sex, whereas eleven studies [5, 7–12, 16–19] had groups matched for age, sex, comorbidity, physical condition factors (BMI), and tumor stage. The matching was also performed in terms of demographic data, tumor characteristics, operative data, and postoperative outcomes (Fig. 1).

**Table 1:** Study characteristics for the included studies

Study ID	Study setting	Total no. of patients		Age (range)		Male: female	
		LAR group	APR group	LAR group	APR group	LAR group	APR group
Chambers 2010 [8]	United kingdom	93	70	67.1 (38.4–86.4)	63.5 (32–83.4)	69:24	39:31
Chuwa 2006 [11]	Singapore	677	93	65 (22–89)	64 (33–93)	392:285	52:41
He 2002 [18]	China	128	356	41.6 (27–61)	58 (35–84)	72:56	214:142
Heald 1997 [16]	United kingdom	105	31	62.7 (27–97)	62.7 (27–97)	NS	NS
Kim 2012 [12]	South Korea	402	402	54 (45–63)	54 (44–64)	238:164	237:165
Law 2001 [17]	Hong Kong	123	57	63.6 (25–83)	65.0 (26–86)	71:52	36:21
Li 2006 [6]	China	244	355	NS	NS	125:119	197:158
Li 2009 [19]	China	53	25	61 (35–79)	65 (43–75)	31:26	19:9
Marr 2005 [9]	United kingdom	355	181	NS	NS	NS	NS
Nagtegaal 2005 [5]	United kingdom	205	453	63.9 (27–85)	64.6 (25–87)	97:108	254:199
Wibe 2004 [15]	Norway	1,315	821	NS	NS	859:456	478:343
Shihab 2010 [7]	United kingdom	81	72	67.3	64.8	54:27	51:21
Campos-Lobato 2011 [10]	USA	85	68	55 (45–61)	63 (54–74)	23:27	26:38

*NS* Not stated, *LAR* Low anterior resection *APR* Abdominoperineal resection

**Table 2:** quality assessment of included studies

Study ID	Adequate sequence generation	Allocation concealment	Blinding	Incomplete outcome data	Free from select reporting bias	Free from other potential sources bias
Chambers 2010 [8]	Unclear	Unclear	Unclear	Yes	Unclear	Unclear
Chuwa 2006 [11]	Unclear	Yes	Unclear	Yes	Yes	Yes
He 2002 [18]	Yes	Yes	Unclear	Yes	Yes	Unclear
Heald 1997 [16]	Yes	Unclear	Unclear	Yes	Unclear	Yes
Kim 2012 [12]	Yes	Yes	Unclear	Yes	Yes	Yes
Law 2001 [17]	Yes	Yes	Unclear	Yes	Yes	Yes
Li 2006 [6]	Yes	Yes	Unclear	Yes	Yes	Yes
Li 2009 [19]	Yes	Yes	Unclear	Yes	Yes	Yes
Marr 2005 [9]	Unclear	Yes	Unclear	Yes	Yes	Unclear
Nagtegaal 2005 [5]	Yes	Yes	Unclear	Yes	Yes	Unclear
Wibe 2004 [15]	Yes	Unclear	Unclear	Yes	Unclear	Yes
Shihab 2010 [7]	Yes	Yes	Unclear	Yes	Yes	Yes
Campos-Lobato 2011 [10]	Yes	Yes	Unclear	Yes	Yes	Yes

### Overall Survival

**Fig. 2:**
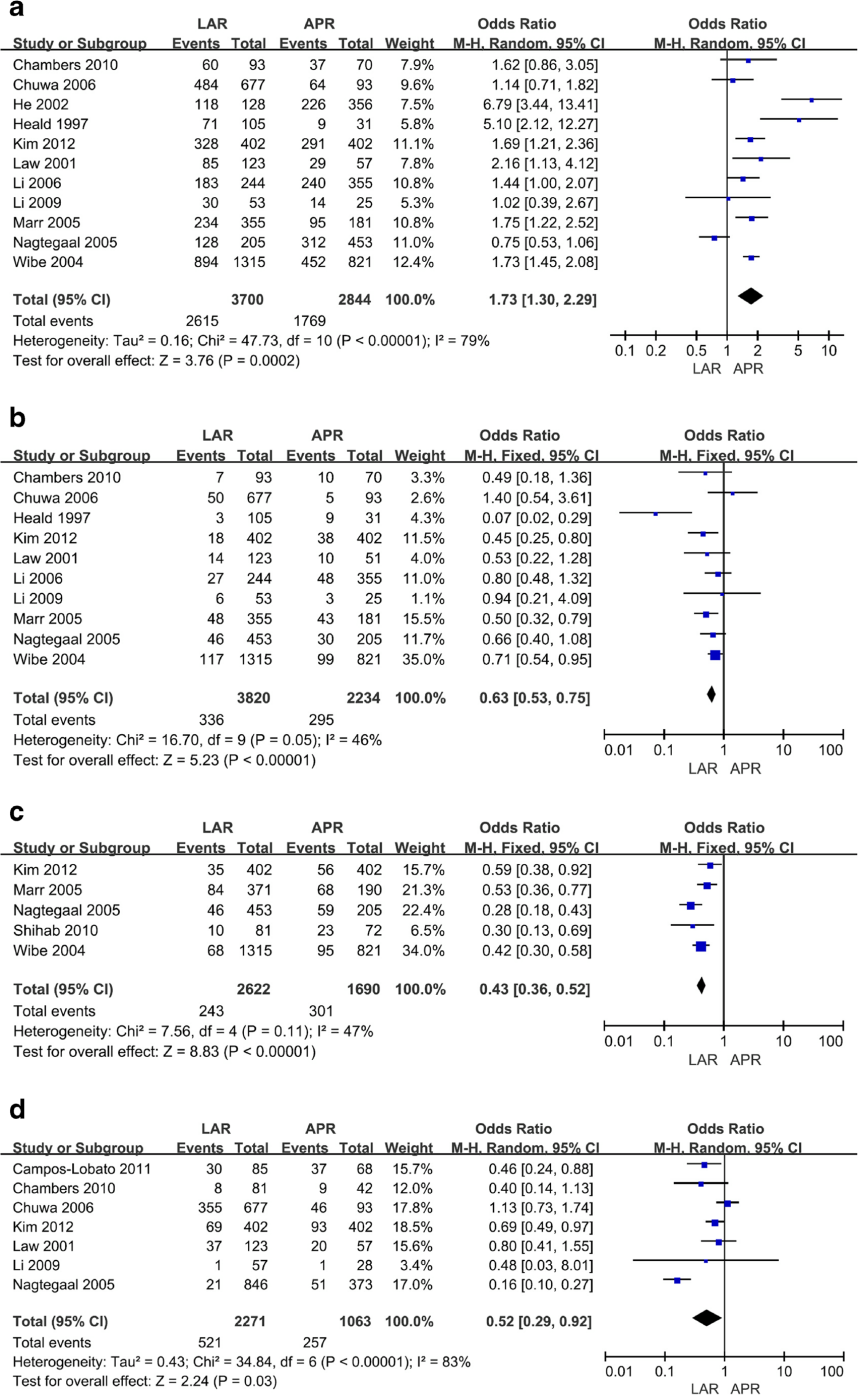
Forest plot of RR for 5-year survival, local recurrence, CRM and complication among included studies. A. results of the meta-analysis on 5-year survival. B. results of the meta-analysis on local recurrence. C. results of the meta-analysis on CRM. D. results of the meta-analysis on complication

As Fig. 2a shows, eleven studies evaluated 5-year survival rate after operation [5, 6, 8, 9, 11, 12, 15–19]. The statistic data was significantly favorable to LAR group at 5-year survival (pooled *OR* = 1.73, 95%CI: 1.30–2.29, *P* = 0.0002 random effect) (*χ* ^2^ = 47.73, *df* = 10, *P* < 0.00001, *I* ^*2*^ = 79 %) (Fig. 2a). Only Nagtegaal et al. [5] (57.6 % versus 38.5 %; *P* = 0.008) showed a better outcome in the APR group.

### Local Recurrence Rate and CRM Rate After Operation

Ten studies evaluated local recurrence rate after operation [5, 6, 8, 9, 11, 12, 15–17, 19], results of the pooled analysis showed that local recurrence rate of APR group was significantly higher than that in LAR group (pooled *OR* = 0.63, 95%CI: 0.53–0.75, *P* < 0.00001 fixed-effect). The results of homogeneity test showed that *χ* ^*2*^ = 16.70, *df* = 9, *P* = 0.05, *I* ^*2*^ = 46 %. The circumferential resection margin (CRM) were high involved in APR group than in LAR group. (5 trials reported the data, pooled *OR* = 0.43, 95%CI: 0.36–0.52, *P* < 0.00001 fixed-effect) (*χ* ^*2*^ = 7.56, *df* = 4, *P* = 0.11, *I* ^*2*^ = 47 %) (Fig 2b and c).

### Overall Complications

It was not possible to compare the urinary retention in the included studies. The reason is that only Campos-Lobato et al. [10] and Kim et al. [12] reported these outcomes in the full article published in 2011 and 2012 respectively. The overall incidence of surgical complications was available, including hemorrhage, ureter injury, bladder injury, and anastomotic rupture. Seven studies reported overall complications [5, 8, 10–12, 17, 19], the results of homogeneity test showed that there was a significant heterogeneity (*χ* ^*2*^ = 34.84, *df* = 6, *P* < 0.00001, *I* ^*2*^ = 83 %) and adopted random-effects model to analyze. Results of the pooled analysis showed that the incidents of overall complications of APR group was higher compared with LAR group (pooled *RR* = 0.52, 95%CI: 0.29–0.92, *P* = 0.03 random-effect) (Fig. 2d).

### Publication Bias

**Fig. 3:**
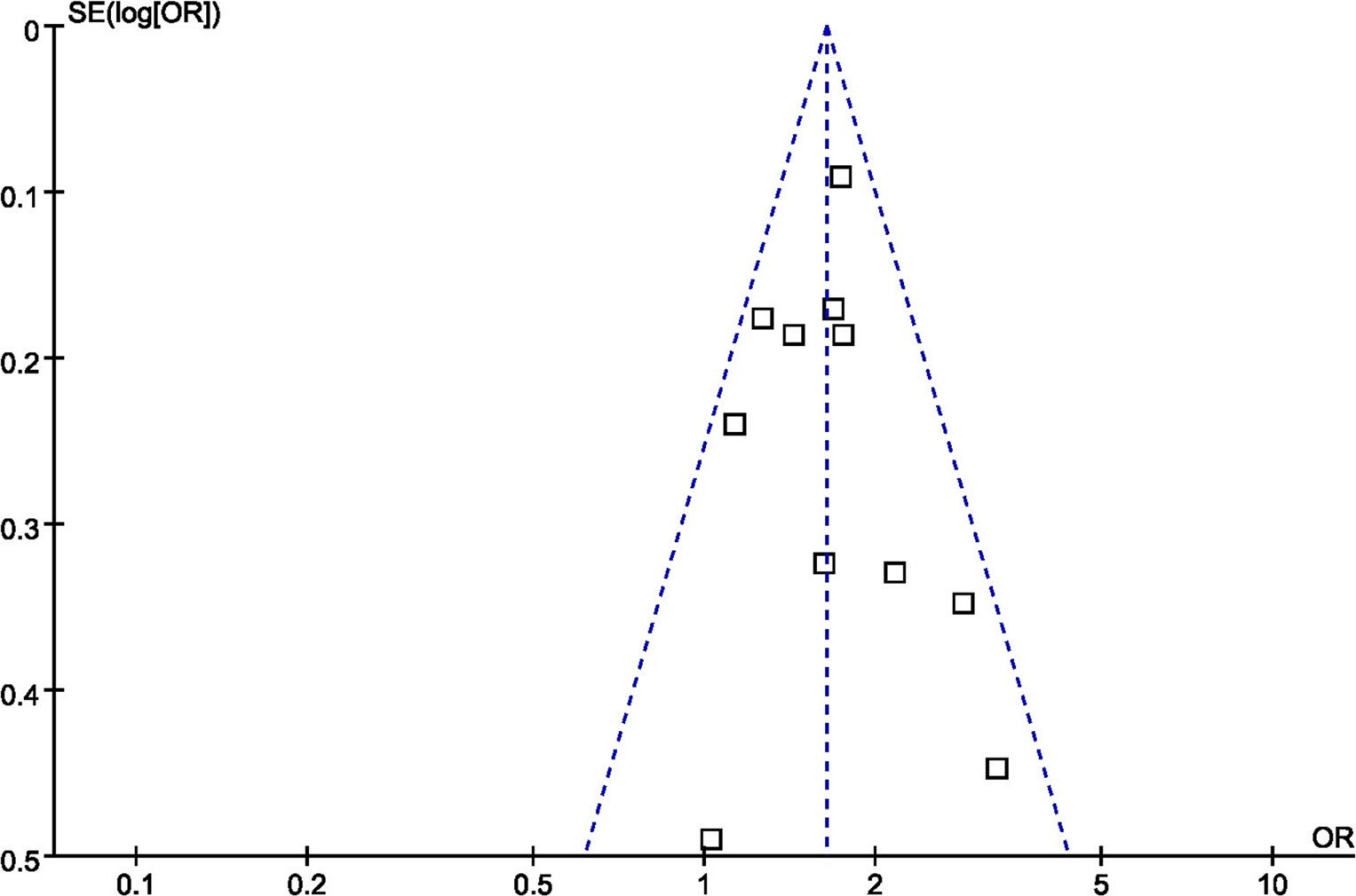
Funnel plot of studies to detect publication bias

The Funnel plot recommended for meta-analyses did not show an asymmetrical pattern (Fig. 3), indicating a publication bias did not exist. Besides, an influence analysis was used to evaluate the influence of single study on the summary effect. The meta-analysis was not dominated by any individual study, and removing any study at a time made no difference.

## Discussion

Abdominoperineal resection (APR) was first described by sir W. Ernest Miles in 1908 [20]. For a century, it had been the gold standard for all cancers of the lower third and bulky tumors of the middle third of the rectum. However, there were significant numbers of patients who do not agree to the treatment with permanent iliac colostomy. Facing the challenge of the physical, psychological consequences of a permanent iliac colostomy from those patients, majority of surgeons tried to develop procedures to save the lower end. The concept of total mesorectal excision (TME) and the increasing use of stapling devices for rectal and anal anastomoses have had a significant impact on the treatment of distal cancer. With the use of TME and a close distal resection margin, the need for sphincter ablation can be reduced. Although APR has been described as standard treatment for tumors less than 8 cm from the anal verge, more patients with distal rectal cancer are now being treated with sphincter-saving surgery. Low anterior resection (LAR) with straight coloanal anastomosis gained wide acceptance in the treatment of the cancer of lower third of the rectum. With the advent of stapling instruments and the techniques such as transanal coloanal anastomosis, the incidence of APR is reduced by 50 %.

For those patients with lower rectal cancer eligible for surgical treatment, whether APR or LAR is the better choice remains controversial. In a study of 608 rectal cancer patients by Marr et al. [9]. APR was associated with greater local recurrence and a lower 5-year survival rate compared with LAR [9]. Two studies reported that the incidence of CRM involvement in APR was more than threefold greater than in LAR [5, 9]. To avoid CRM involvement, Holm et al. recommend an extended APR, which includes en bloc excision of the levator muscles with the anus and the lower rectum [21]. However, another reasearch showed that the oncologic outcomes of patients treated by APR are not worse than those treated by LAR [11]. Kim and colleagues indicated that APR can be used safely without impairing oncological outcome when performed with appropriate skill to achieve R0 resection [12]. These conflicting results were likely due to small sample size of the study. Meta-analysis was originally developed to combine the results of randomized controlled trails, and recently this approach has been applied successfully for identification of prognostic indicators in patients with malignant diseases [22–24].

This meta-analysis is the first study to systematically estimate the technical feasibility, effectiveness, and safety of APR and LAR in the treatment of lower rectal cancer, through a systematic review of published comparative studies. The results of this meta-analysis indicated that APR led to worse cancer specific outcome than LAR. In other words, APR group could not increase 5-year survival rate and reduce operative complications compared with LAR, in keeping with other published results [16].

The tumors were lower and larger in the APR group, although the patients in the LAR and APR groups were comparable in terms of age, tumor stage, and neoadjuvant treatment. The distribution of tumor stage within the APR and LAR groups was similar for both patient, however, it would not be possible to avoid this bias as bigger tumors would tend to undergo an APR, as sphincter saving would not be attempted. The extent of tumor spread in itself is therefore unlikely to account for the increased CRM involvement, increased local recurrence, and poor survival in the APR group [9].

Circumferential resection margin (CRM) involvement is a strong prognostic indicator for local recurrence. The patients who underwent APR had a significantly higher incidence of circumferential margin involvement, which is associated with both local recurrence and poor survival. There is evidence to suggest that APR is associated with a higher CRM involvement rate, which has been found to be an acceptable surrogate endpoint for increased local recurrence and lower patient survival [25]. CRM involvement increases the more distally the tumor is located [26]. One analysis [27] showed that CRM is of prognostic value for both local recurrence and overall survival in patients treated with an APR, similar to previously published results demonstrating the importance of CRM for all patients [26]. It can be concluded that the poor prognosis of APR was attributable to frequent CRM involvement. Possible reasons include higher incidence of inadequate excision in APR, or that lymph node involvement may follow a different pattern in low rectal carcinomas. Furthermore, distance from the anal verge was related to the completeness of mesorectal excision. Just as how chuwa et al. [11] had pointed out that only 37 %of the patients whose tumors were located 5 cm or less from the anal verge had complete mesorectal excision, because of the greater difficulty of performing a perfect TME low down in the pelvis. And TME cannot always be performed down to the levators in APR because of the presence of a large tumor around this level. The frequency of CRM involvement for APR has not diminished with TME. CRM involvement in the APR specimens is related the removal of less tissue at the level of the tumor in an APR [9]. The poor prognosis of the patients with an APR is ascribed to the resection plane of the operation leading to a high frequency of margin involvement by tumor and perforation with this current surgical technique [26].

Local recurrence is an important indicator of the success of rectal surgery. The high rates of local recurrence of APRs could be explained by a number of factors either singly or in combination. Although there is convincing evidence that TME reduces local recurrence rates by 1–6 % [9], APR may be associated with a different pattern of lymphatic spread, which is not included in the “tumor package” excised by TME, or inadequate surgical resection may occur in a higher proportion of patients. Inadequate excision appears to be the major factor determining outcome, but APR, the form of more radical surgery, may not improve the current situation of high local recurrences and poorer survival.

Despite important progress made in the past decade regarding techniques and perioperative management, patients with rectal cancer still inevitably experience surgical complications. The commonest complication of APR was perineal wound failure, and anastomotic leak was the commonest complication after LAR [8]. With the lowering level of colo-anal anastomosis and increasing demands for anal-sphincter preservation, risks such as anastomotic leakage and hemorrhage are considered to be the major complications of LAR. And Jorge et al. [28] reported a leakage rate of 11-12 % following rectal cancer surgery. However, we have demonstrated that the postoperative complications such as pelvic abscess, voiding difficulty, sexual dysfunction, erectile dysfunction and ejaculatory dysfunction, is more higher in the APR group than in the LAR group. Besides, only 20.9 % of patients in the APR group satisfied with the permanent stoma [12]. Patients undergoing APR do have some restriction in their postoperative QoL, such as body image, which can be severely disruptive to their social life. The results of meta-analysis indicated that the incidence of LAR complications was higher compared with APR group. These data are in line with the incidence reported in literature.

Sphincter-preserving surgery must be considered the primary procedure of choice, LAR can be safely used in patients with proper technique without impairing oncological outcome, Although an APR is necessary in many patients with low or advanced tumors and cannot be substituted with an LAR.

Certain limitations in the present meta-analysis need to be pointed out. First, this meta-analysis contain only retrospective data, all the studies included were observational, and the small number of cases in several studies also decreased the reliability of the results, which made it difficult to acquire strong evidence for the conclusions. Although we compared the study groups with respect to all variables known to affect the primary outcome, there are certainly confounders and variables unaccounted for that may affect the results. There is a conspicuous absence of prospective randomized trials in this subject area, which should be addressed in future research. Second, the studies included in the analysis were mostly conducted at major institutions. Therefore, the patients evaluated might not reflect patient populations in the community. In other words, the evaluation of data coming from high volume centers might not represent the real world. Third, heterogeneity between studies was low for most of the dichotomous variables examined in this analysis, but was marked for all the continuous variables. There was significant variability in terms of definitions, inclusion criteria, exclusion criteria, operating technique, and measurement of outcomes. It was not possible to match all patient groups for age, BMI, tumor stage, preoperative therapy, and previous abdominal history. All these factors may have contributed to the high heterogeneity between studies. Use of the RE model for pooled data might minimize the effects of heterogeneity, but does not abolish them. The degree of heterogeneity fell for most outcomes with sensitivity analysis, but this difference was not significant. Fourth, some perioperative data reported as median (range) were calculated to reach the mean (SD) values with the techniques introduced in literature [29], and it is considered a limitation of the daa analysis in the current study. Fifth, we excluded the patients accepted other treatment before and/ or after surgery. However, preoperative radiotherapy, chemotherapy, and/or neoadjuvant chemoradiation of advanced rectal cancer could be potentially be useful for local control and sphincter saving [30]. Finally, some authors did not report the proportion of patients lost to follow-up, which may influence the reliability of the conclusions. Therefore, we tried to optimize standardization, but some remaining variability in definitions was unavoidable, and there were subtle differences in assessment of primary outcomes between studies. We tried to overcome this potential issue by defining low rectal cancer as a distal tumor margin within 6 cm of the anal verge and by including only those studies that included patient details that allowed us to best apply our definitions.

The present meta-analysis suggests that LAR has a higher 5-year survival rate, low CRM rate, local recurrence and complications rate than APR. in selected low rectal cancer patients, LAR is a better option than APR. Despite our rigorous methodology, the inherent limitations of the included studies should be considered, and conclusions drawn from our pooled results should be interpreted with caution. Future prospective, multicentre, and randomized trials including small number of cases, preoperative radiotherapy, chemotherapy, and/or neoadjuvant chemoradiation administered to the patients will be useful to confirm this conclusion.
